# A robust radiomic-based machine learning approach to detect cardiac amyloidosis using cardiac computed tomography

**DOI:** 10.3389/fradi.2023.1193046

**Published:** 2023-06-16

**Authors:** Francesca Lo Iacono, Riccardo Maragna, Gianluca Pontone, Valentina D. A. Corino

**Affiliations:** ^1^ Department of Electronics, Information and Bioengineering, Politecnico di Milano, Milan, Italy; ^2^ Department of Perioperative Cardiology and Cardiovascular Imaging, Centro Cardiologico Monzino IRCCS, Milan, Italy; ^3^ Department of Biomedical, Surgical and Dental Sciences, University of Milan, Milan, Italy

**Keywords:** cardiac amyloidosis, aortic stenosis, radiomics, radiomic feature stability, cardiac computed tomography

## Abstract

**Introduction:**

Cardiac amyloidosis (CA) shares similar clinical and imaging characteristics (e.g., hypertrophic phenotype) with aortic stenosis (AS), but its prognosis is generally worse than severe AS alone. Recent studies suggest that the presence of CA is frequent (1 out of 8 patients) in patients with severe AS. The coexistence of the two diseases complicates the prognosis and therapeutic management of both conditions. Thus, there is an urgent need to standardize and optimize the diagnostic process of CA and AS. The aim of this study is to develop a robust and reliable radiomics-based pipeline to differentiate the two pathologies.

**Methods:**

Thirty patients were included in the study, equally divided between CA and AS. For each patient, a cardiac computed tomography (CCT) was analyzed by extracting 107 radiomics features from the LV wall. Feature robustness was evaluated by means of geometrical transformations to the ROIs and intra-class correlation coefficient (ICC) computation. Various correlation thresholds (0.80, 0.85, 0.90, 0.95, 1), feature selection methods [*p*-value, least absolute shrinkage and selection operator (LASSO), semi-supervised LASSO, principal component analysis (PCA), semi-supervised PCA, sequential forwards selection] and machine learning classifiers (k-nearest neighbors, support vector machine, decision tree, logistic regression and gradient boosting) were assessed using a leave-one-out cross-validation. Data augmentation was performed using the synthetic minority oversampling technique. Finally, explainability analysis was performed by using the SHapley Additive exPlanations (SHAP) method.

**Results:**

Ninety-two radiomic features were selected as robust and used in the further steps. Best performances of classification were obtained using a correlation threshold of 0.95, PCA (keeping 95% of the variance, corresponding to 9 PCs) and support vector machine classifier reaching an accuracy, sensitivity and specificity of 0.93. Four PCs were found to be mainly dependent on textural features, two on first-order statistics and three on shape and size features.

**Conclusion:**

These preliminary results show that radiomics might be used as non-invasive tool able to differentiate CA from AS using clinical routine available images.

## 1. Introduction

Cardiac amyloidosis (CA) is an increasingly diagnosed condition caused by conformational changes in amyloidogenic proteins leading to amyloid fibril deposition in the heart (1). The two predominant amyloid proteins found in the heart are immunoglobulin light chain (AL) and transthyretin (ATTR) (2). ATTR-CA occurs in two most common forms: wild-type, or senile, transthyretin (wtATTR), more prevalent in elderly people, and hereditary or variable TTR (vATTR), genetic autosomal dominant disease (3). Clinical presentation associated to CA shows an increased biventricular wall thickness, myocardial stiffening and restrictive physiology of the left and right ventricles (2) caused by amyloid fibril deposition in the extracellular myocardial space. Similar myocardial remodeling processes affect the heart of patients with aortic stenosis (AS). AS is the most common cause of valvular heart disease (4). It is characterized by a progressive aortic valve narrowing leading to an increase in afterload and wall stress, compensated with a gradual hypertrophy of left ventricle (LV) (5). Therefore, as CA shares several common phenotypical features with AS, and considering the high prevalence of subclinical CA among AS undergoing transcatheter aortic valve implantation (TAVI), the differential diagnosis of these two entities has important prognostic and therapeutic implications. Patients affected by both CA and AS have, indeed, worsen prognosis and are less likely to benefit from aortic valve replacement (AVR) surgery with respect to lone AS patients. In addition, the recent introduction of new effective therapies for improving the prognosis of patients with CA makes early recognition of this pathology even more relevant than in the past.

In this framework, radiomics can be a non-invasive tool useful to perform differential diagnosis starting from medical images such as cardiac computed tomography (CCT), usually used for interventional planning of AS patients undergoing TAVI. Radiomics is an emerging research field aimed to improve diagnosis, characterization, and prognosis using quantitative features extracted from medical images. Radiomics is widely employed in oncology for tumors characterization (6, 7), treatment response (8–10), and overall survival analysis (11, 12). Recently, it has been proposed in the cardiovascular field to improve diagnostic accuracy, patients cardiac risk prediction and stratification (13–22).

As radiomics generates hundreds of features, a crucial step of radiomic workflow is selecting features according to their relevance with respect to the clinical question of interest. Assessing features robustness is a necessary preliminary step in the process of feature selection (23). The most applied techniques to evaluate features reliability are test-retest and multiple delineations of the ROI (15, 17, 18), methods affected by a time-consuming intrinsic limit: test-retest requires multiple scan acquisitions, whereas multiple delineations require several ROI segmentations. To overcome these problems, in the current study, for the first time in CCT, features robustness was assessed performing geometrical transformations of the ROIs (23). To mimic multiple manual delineations, small ROI transformations are applied to simulate errors due to manual delineation thus assessing feature stability. In addition, large ROI transformations are performed to assess feature discrimination capacity. The underlying hypothesis is that robust features need to be stable, i.e., similar for small transformations, and discriminative, i.e., different for large transformation. Studies on feature robustness already exist in oncological field (23–25), whereas a very limited research is available in cardiovascular radiomics (15, 26).

In this study, we aim to develop a robust and reliable pipeline to differentiate between hypertrophic phenotype due to CA versus AS using radiomics and machine learning techniques. The final goal is to provide early detection of CA patients to administer specific treatment and improve their prognosis. Radiomic features will be extracted from the LV muscle in CCT, and different methods of feature selection and machine learning algorithm will be tested.

## 2. Methods

### 2.1. Study population and baseline characteristics

Thirty patients were included in the study: 15 with CA and 15 with AS. CA was defined by the presence of ATTR amyloid in a myocardial biopsy (Congo red and immunohistochemical staining) or positive DPD scintigraphy, while AL amyloidosis was proven with biopsies from non-cardiac tissues. In AS patients, the concomitant presence of CA was excluded by bone scintigraphy and/or cardiac magnetic resonance (CMR).

All patients underwent comprehensive evaluation with transthoracic echocardiography using commercially available equipment (iE33 or Epiq, Philips Medical System, or Vivid-9, GE Healthcare) measuring LV end-diastolic (LVEDV) and end-systolic (LVESV) volumes indexed for body surface area, LV ejection fraction (LVEF) and intraventricular septum thickness (IVS). Clinical characteristics are shown in Table 1.

**Table 1:** Baseline characteristics of study population.

	All (*n* = 30)	AS (*n* = 15)	CA (*n* = 15)	*p* value
Age, years	78 (70–83)	82 (79–85)	71 (65–77)	*p* < 0.01
Female	13 (43%)	4 (27%)	9 (60%)	ns
Body surface area, m^2^	1.9 (1.8–2.0)	1.9 (1.8–1.9)	1.9 (1.8–2.1)	ns
Body mass index, kg/m^2^	27 (25–31)	29 (26–31)	26 (22–32)	ns
LVESVi, ml/m^2^	19 (13–24)	21 (15–24)	18 (14–24)	ns
LVEDVi, ml/m^2^	47 (37–63)	58 (43–65)	38 (30–57)	ns
LVEF, %	58 (46–68)	64 (60–72)	51 (41–58)	*p* < 0.01
IVS thickness, mm	13 (12–16)	13 (13–14)	15 (12–17)	ns

Values are expressed as absolute number and percentage or median and interquartile range. LVEDVi, left ventricle end-diastolic volume index; LVEF, left ventricle ejection fraction; LVESVi, left ventricle end-systolic volume index; IVS, intraventricular septum; ns, not significant.

The institutional Ethical Committee approved the study, and all the patients signed the informed consent.

### 2.2. CCT scan protocol

CCT examinations were performed using Revolution CT (GE Healthcare, Milwaukee, WI) or Aquilion ONE VisionTM (Canon Medical Systems Corp., Tokyo, Japan). Details of the CT image acquisition parameter are reported in Supplementary Table S1.

No premedication with beta-blockers or nitrates was added before CT acquisition. Patients received a fixed dose of 50 ml bolus of contrast medium (400 mg of iodine per milliliter, Iomeprol; Bracco, Milan, Italy) despite the BMI via an antecubital vein at an infusion rate of 5 ml s^−1^ followed by 50 ml of saline solution at 5 ml·s^−1^.

### 2.3. Images segmentation, preprocessing and radiomics features extraction

For each patient, the LV wall was manually segmented by a unique operator, and all the segmentations were revised by a different, blinded, level III, European Association of Cardiovascular Imaging certified operator (27).

Image preprocessing was performed to reduce the imaging-related variability: a 3D Gaussian filter with a 3 × 3 × 3 voxel kernel and *σ *= 0.5 was used to denoise the images. Then, voxel size resampling to an isotropic resolution of 2 mm [as in (28)] was performed with B-spline interpolation.

One-hundred and seven radiomic features were extracted using Pyradiomics 3.0 (29). The features belong to the following classes: shape and size (SS, 14 features), first order statistics (FOS, 18 features) and textural features (75 features). See Pyradiomics documentation for more details (29). A fixed-bin width histogram discretization (0.5 Hounsfield units per bin) was used prior to features extraction. Since features were extracted with Pyradiomics, they were compatible with the Image Biomarker Standardization Initiative (30).

### 2.4. Feature selection

All the feature selection steps were performed on the training set and then applied on the test set.

#### 2.4.1. Selection of stable and discriminant features

Stability and discrimination capacity were assessed using geometrical transformations (translations) of the ROIs as in (23). The entire workflow is implemented in MATLAB 2017a (Mathworks, Natick, MA, USA) and applied to FOS and textural features, as SS features do not change with a ROI translation.

**Figure 1:**
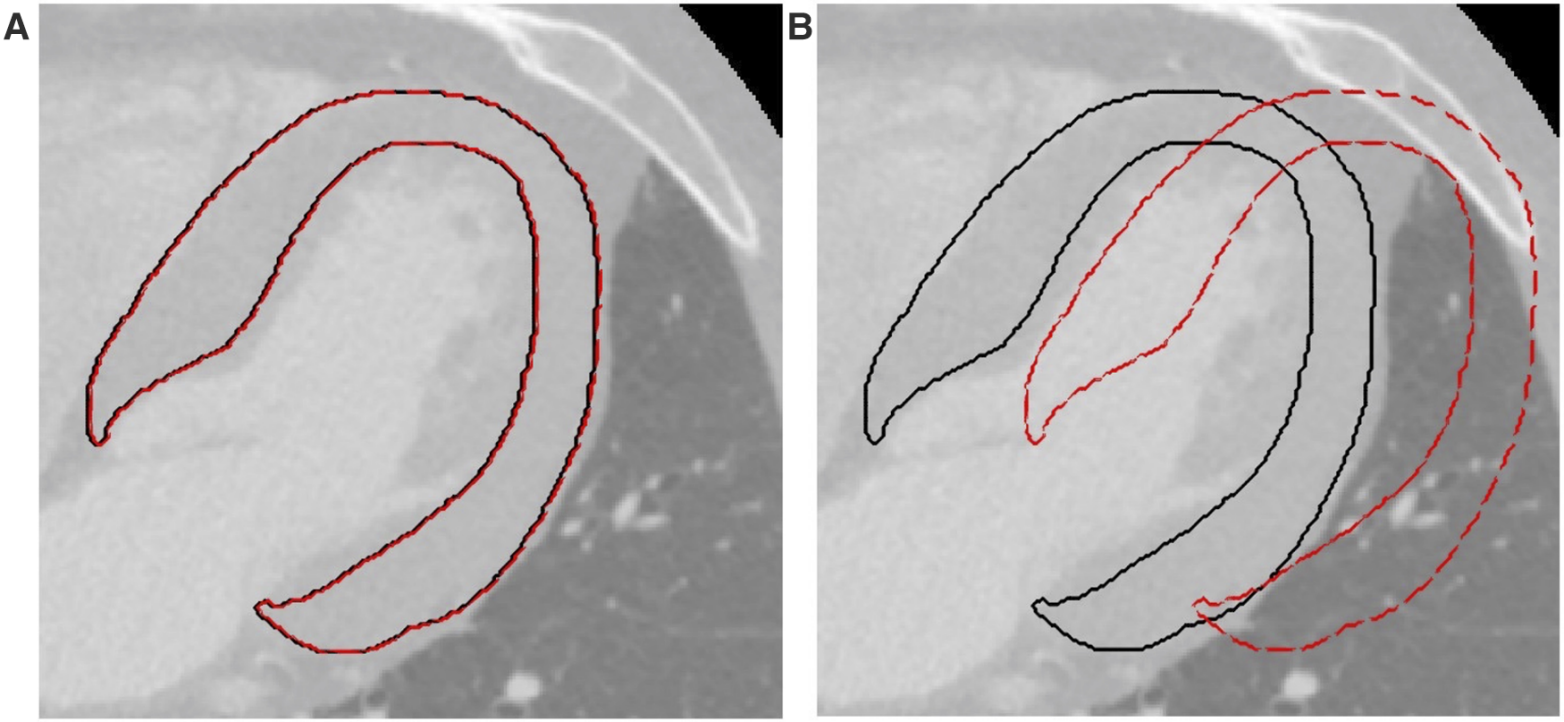
Example of translations applied to the ROIs. (**A**) Minimal entity translation: dashed red line represents a translation of 0.5% in the *x* direction. (**B**) Maximal entity translation: dashed red line represents a translation of 30% in the *x* direction. In both figures, continuous black lines represent the contours of the original ROIs. ROI, region of interest.

Two entity translations were applied to the ROIs, along both the *x* (medial-lateral) and *y* (antero-posterior) directions. The minimal entity translation was ±0.5% the length of the bounding box surrounding the ROI in the direction of interest (Figure 1A), and the maximal entity translation was ±30% (Figure 1B). Radiomic features were computed on each translated ROI and compared to the ones obtained from the original ROI by expert cardiac radiologists. Briefly, for each feature calculated from a single ROI, 4 intraclass correlation coefficients (ICCs) with their mean (ICC_mean_) were calculated. Following the general guidelines (31), two ICC threshold values were identified to select robust features: ICC = 0.75, which indicates good agreement between data, and ICC = 0.5, which reflects poor similarity. Thus, a feature is considered stable if the ICC_mean_ for the minimal entity translation is higher than 0.75 and discriminative if the ICC_mean_ for the maximal entity translation is lower than 0.5. For each translation, the percentage of overlapping volume was also computed to assess whether most of the original ROI was part of the transformed ROI. After this step, the features were *z*-scored.

#### 2.4.2. Selection of non-redundant features

The second step consisted of a correlation-based feature selection, performed to ensure a set of features with low internal redundancy. When a pair of features had an absolute Spearman correlation coefficient above a fixed threshold only one of the two was kept. In particular, the one with lower mean correlation with all the other *n*-2 features was selected. Obviously, the higher the threshold, the fewer features were removed. Five thresholds were considered (0.80, 0.85, 0.90, 0.95, 1), where a threshold equal to 1 means no features are removed in this step.

#### 2.4.3. Selection of more relevant features

Six feature selection methods were tested (32), namely: *p*-value, least absolute shrinkage and selection operator (LASSO), semi-supervised LASSO (ssLASSO), principal component analysis (PCA), semi-supervised PCA (ssPCA) and sequential forwards selection (SFS). Briefly, the *p*-value method performs a Wilcoxon rank-sum test on each feature to identify the ones significantly different between the two unpaired groups of patients; LASSO shrinks the coefficients of less important features to zero to keep only the most important features by mean of penalty factor lambda; ssLASSO employs statistically significant features (selected by the *p*-value method) as input for LASSO algorithm; the PCA transforms the high-dimensional dataset into a lower-dimensional space by identifying the most important principal components capturing the maximum variance in the data, while minimizing the loss of information; ssPCA employs statistically significant features (selected by the *p*-value method) as input for PCA; SFS iteratively adds the most relevant features to the model, one at a time, based on their individual contribution to the model's performance (until the score improvement exceeds a threshold, here fixed to 0.02).

### 2.5. Classification model development

The dataset was divided into training and test sets using a leave-one-out (LOO) cross-validation method, as a result, for each iteration, the test set contained only one observation, while the others were used as training. Since the dataset was balanced but small, data augmentation was performed on the training set, that was doubled in size (maintaining the class balance) using the synthetic minority oversampling technique (SMOTE) algorithm (33). SMOTE over-samples the minority class by generating synthetic examples. This is done by selecting each sample from the minority class and creating new samples along the line segments that connect the sample to its Q nearest neighbors within the minority class. For every minority class sample, we find the Q closest neighbors belonging to the same class and select one of them randomly. The synthetic sample is then positioned at a random point along the line that connects the two original samples. In this study, the value of Q was set to 5. By applying SMOTE, both classes end up having an equal number of instances available for classification. As in this study the classes were balanced, the samples of a single class were first duplicated and subsequently, SMOTE was applied: this procedure was performed separately for both classes and the new samples were added to the original dataset. As a result, the number of training samples was increased from 29 to 58 patients.

To find the best classification model, five machine learning models were trained: k-nearest neighbors (kNN), support vector machine (SVM), decision tree (DT), logistic regression (LR), and gradient boosting (GB). Parameters values set for each classification model are shown in Supplementary Table S2. The *p*-value feature selection method was used to select the best-performing model. Model performance was evaluated based on sensitivity, specificity and diagnostic accuracy, being the latter used to identify the best model. The best pair of feature selection method and correlation threshold was also selected.

**Figure 2:**
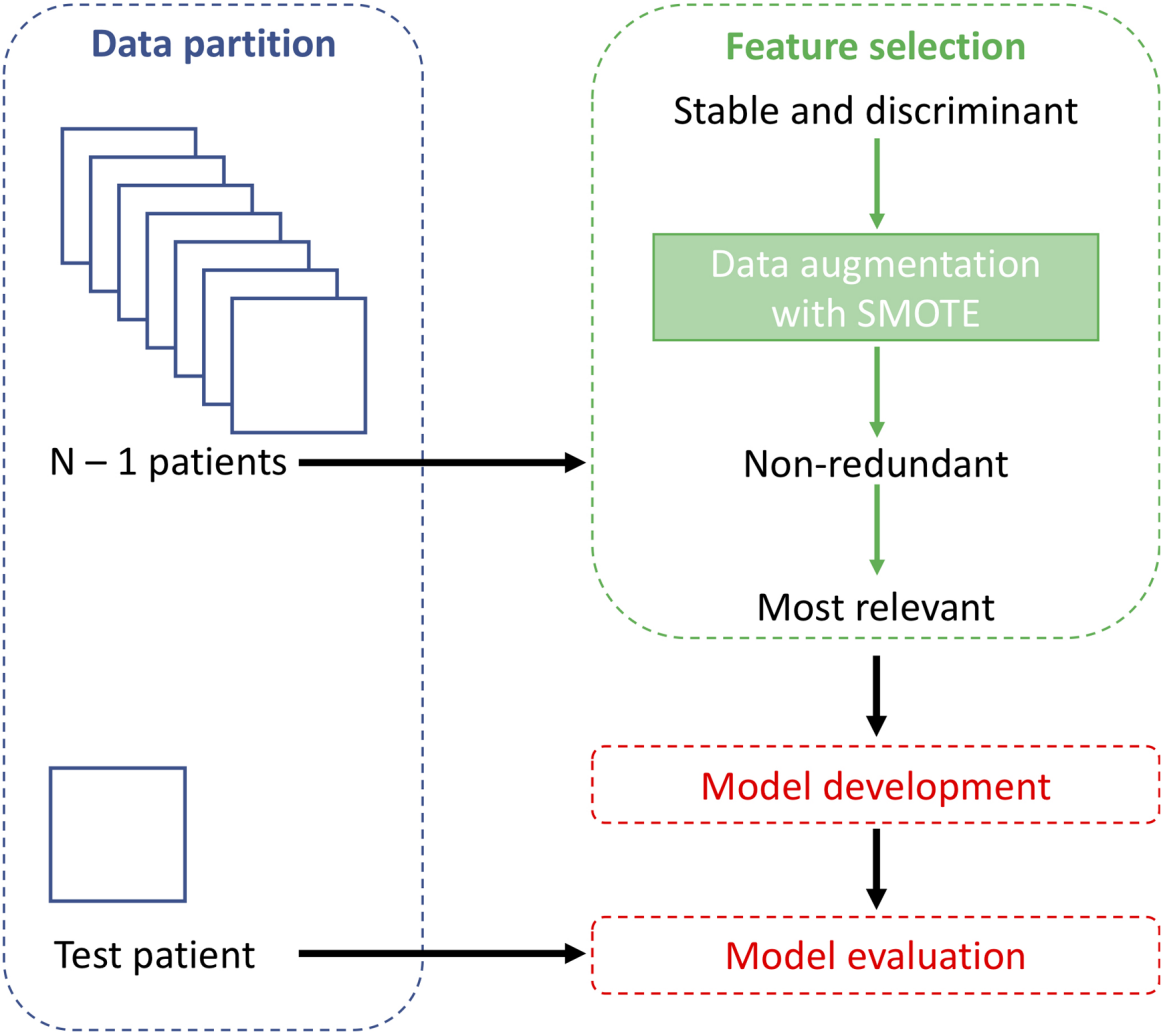
Machine learning workflow.

The entire workflow is shown in Figure 2. The feature selection and classification are implemented in Scikit-Learn Python library.

### 2.6. Explainability analysis

After selecting the best correlation threshold, feature selection method and machine learning model, the final model was built by training on the whole original dataset. As during model development, the training set was oversampled using SMOTE to double the total number of samples.

To interpret the machine learning model and its prediction, the SHapley Additive exPlanations (SHAP) method (34) was used on the best performing model. The SHAP method provides a way to determine the importance of different features in making a prediction by measuring how much each feature contributes to the final prediction. This allows to gain insight into how the model is making its predictions and which features are most influential.

The SHAP method is based on the concept of Shapley values, i.e., the average expected marginal contribution of each feature to the overall prediction. The Shapley values can be visualized using a variety of plots, such as a summary plot or a force plot for single instance, which can help to interpret the results and identify which features are most important in making a prediction.

## 3. Results

### 3.1. Feature selection

**Figure 3:**
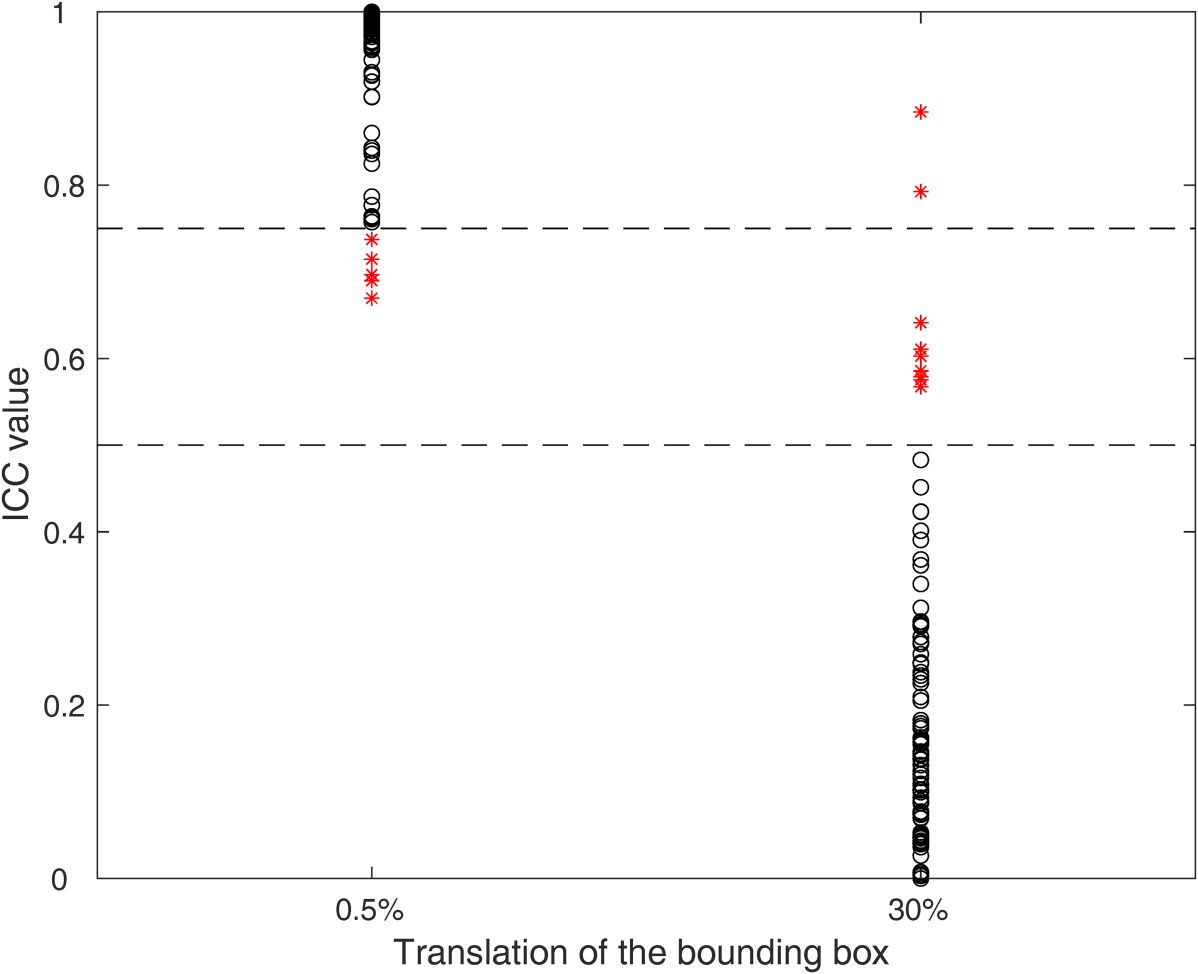
ICCs for the minimal (0.5%) and maximal (30%) entity translation. Each black circle represents a feature satisfying the criterium, while red asterisks represent features not satisfying the criteria. The dashed lines represent the threshold values (0.75 and 0.5) for minimal and maximal entity translations, respectively.

The first step of feature selection identified 92 stable and discriminant features: 6 and 9 features were excluded as they do not satisfy the criterium for the minimal and maximal entity translation, as shown in Figure 3.

The correlation-based feature selection identified on average 23 ± 1, 28 ± 1, 35 ± 1, 47 ± 1 and 92 ± 0 features for the five correlation thresholds (0.80, 0.85, 0.90, 0.95, 1), respectively.

The different feature selection methods selected a number of features ranging from 7 to 58, as shown in Table 2, again depending both on the method itself and the correlation threshold.

**Table 2:** Number of features selected by the different feature selection methods as a function of the used correlation threshold (mean ± standard deviation).

Feature selection method	Correlation threshold				
	0.80	0.85	0.90	0.95	1
*p*-value	14 ± 2	18 ± 2	22 ± 2	30 ± 2	58 ± 4
LASSO	10 ± 2	10 ± 2	11 ± 1	12 ± 1	13 ± 2
ssLASSO	8 ± 2	9 ± 2	10 ± 1	10 ± 2	12 ± 2
PCA	8 ± 0	9 ± 0	9 ± 0	8 ± 0	8 ± 1
ssPCA	7 ± 0	7 ± 0	8 ± 0	8 ± 0	7 ± 0
SFS	11 ± 0	13 ± 0	17 ± 0	23 ± 0	46 ± 0

LASSO, least absolute shrinkage and selection operator; ssLASSO, semi-supervised LASSO; PCA, principal component analysis; ssPCA, semi-supervised PCA; SFS, sequential feature selection.

### 3.2. Influence of the model

**Figure 4:**
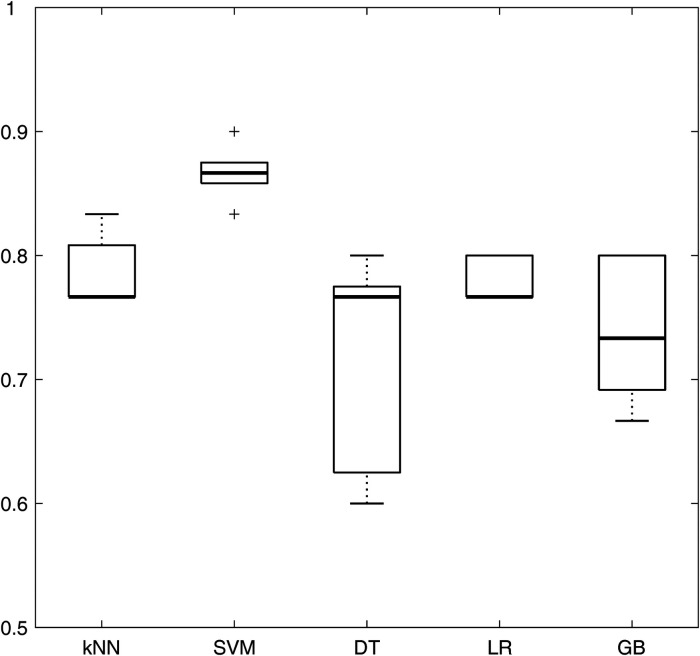
Boxplots representing the average accuracy of the five machine learning models, obtained averaging all the correlation thresholds and *p*-value as feature selection method. kNN, k-nearest neighbors; SVM, support vector machine; DT, decision tree; LR, logistic regression; GB, gradient boosting.

The performance of the five models (namely, kNN, SVM, DT, LR, and GB) in terms of accuracy (averaging the results obtained from the different correlation thresholds) is shown in Figure 4, with respect to the feature sets selected by the *p*-value method. The analogous figures for the other feature selection methods are shown in Supplementary Figure S1, while the other performance metrics are shown in Supplementary Figures S2, S3 for all the feature selection methods. SVM presents the highest median accuracy for any feature selection method and a relatively stable behavior over the correlation thresholds, as highlighted by the small interquartile range (IQR), outperforming all the other models. Considering that SVM model achieved the best performances among different machine learning models with all the feature selection methods, it was selected as the best model to evaluate the influence of the correlation thresholds and feature selection methods. Thus, with the data at disposal, SVM was indicated as the optimal model, trading off complexity and performance.

### 3.3. Influence of the correlation threshold and feature selection

**Figure 5:**
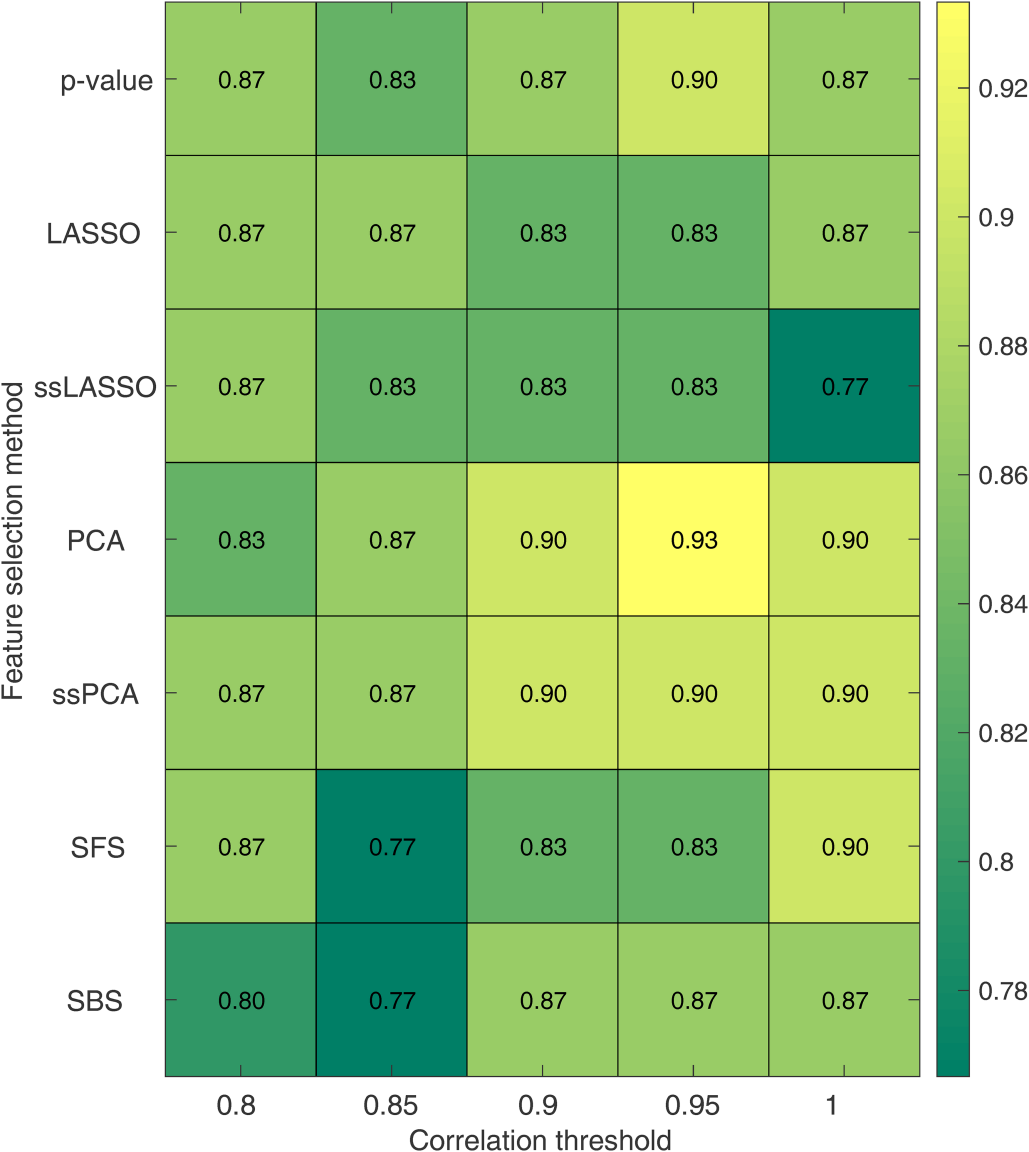
Accuracy for the SVM model as a function of the correlation threshold and the feature selection method. Each row represents a feature selection method, while each column represents a correlation threshold. LASSO, least absolute shrinkage and selection operator; ssLASSO, semi-supervised LASSO; PCA, principal component analysis; ssPCA, semi-supervised PCA; SFS, sequential feature selection.

The impact of the correlation threshold and feature selection method on accuracy is depicted in Figure 5. In the figure, the rows represent different feature selection methods while the columns represent varying correlation thresholds. The bright colors in the color map indicate higher accuracy, while darker colors represent lower accuracy. It is evident that PCA-based methods (both PCA and ssPCA) have higher accuracy across all correlation thresholds, ranging from 0.83 to 0.93 vs. 0.77 to 0.90 for the other feature selection methods. The best performances were observed using the PCA feature selection method which reached an accuracy of 0.90, with no correlation (threshold = 1), and an accuracy of 0.90 and 0.93 with a correlation threshold of 0.90 and 0.95, respectively. The corresponding sensitivity and specificity are shown in Supplementary Tables S3, S4.

### 3.4. Final model and explainability

**Figure 6:**
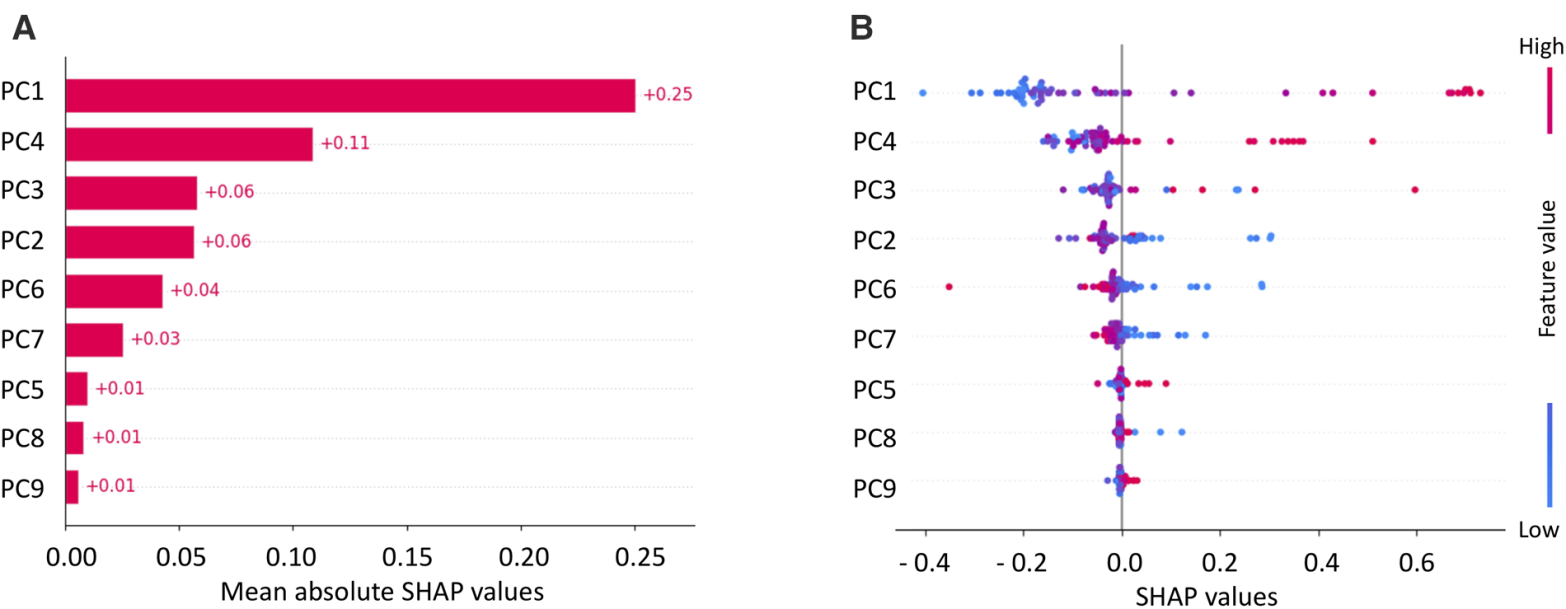
Mean SHAP values for principal components (PCs) of SVM (**A**) and SHAP values with feature values (**B**).

**Figure 7:**
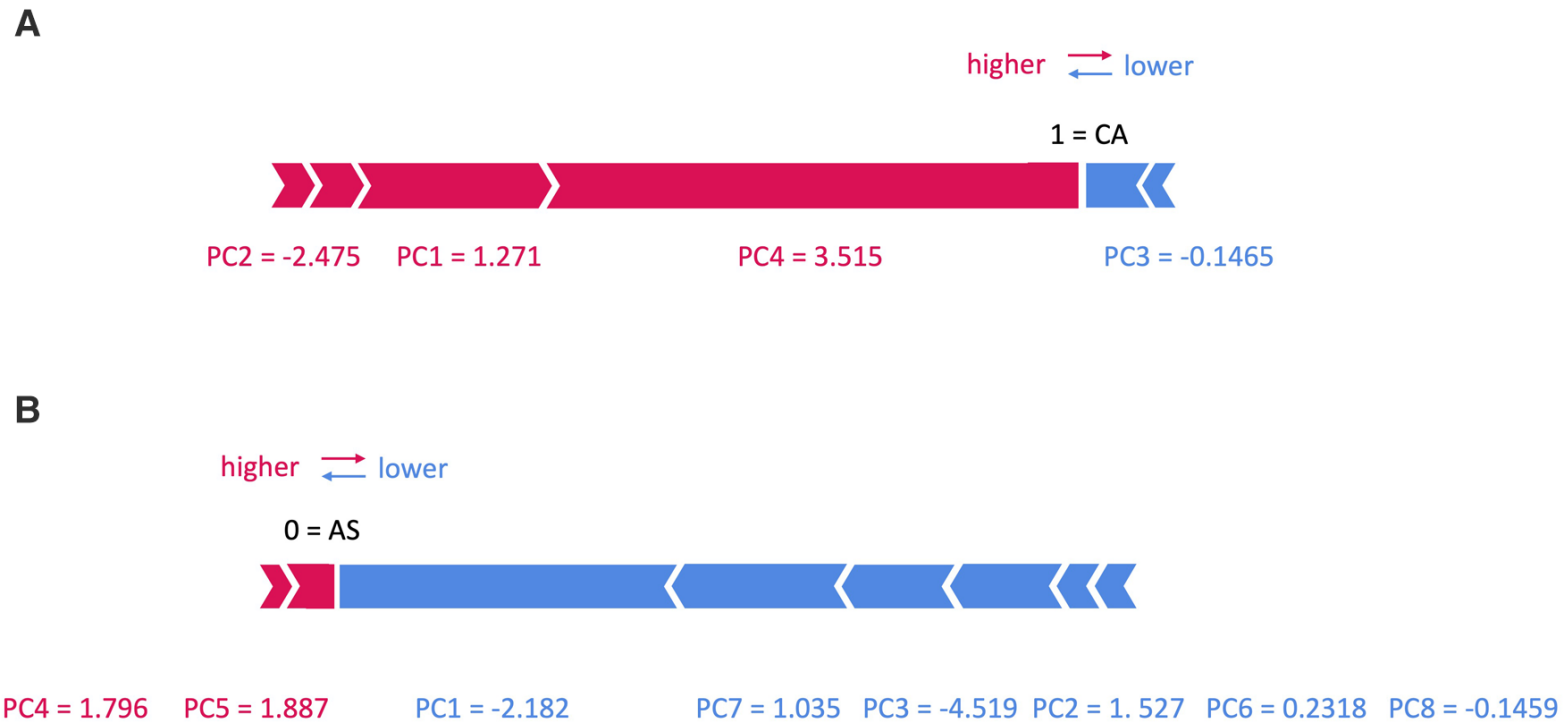
SHAP force plots for two patients with (**A**) cardiac amyloidosis (CA) and (**B**) aortic stenosis (AS).

The final model was built using the model and parameters which were the best performing ones in the LOO cross-validation: SVM, PCA, a correlation threshold of 0.95 and SMOTE. For this model, the explainability by using SHAP analysis was performed, and results are shown in Figures 6, 7. Figure 6A shows the SHAP feature importance for the 9 principal components of the final model. Among them, the first principal component (PC) contributed the most to the model (0.25 mean absolute SHAP value), the next highest-ranking features were the PC 4, 3, 2, 6 and 7, whereas PC1, 5, 8 and 9 contributed less to the model (0.1 mean absolute SHAP value). Figure 6B shows the overall correlation and directionality between features and the SHAP value during model training. Each dot represents one patient, and the color reflects the high and low values of each feature, with the red color indicating a higher value and the blue color indicating a lower value. The *x*-axis of the graph represents the SHAP value, and a positive SHAP value indicates that it contributes positively to predicting CA and vice versa.

In Figure 7, two examples are shown: in panel (A) an example of a patient with CA is shown, with a prediction equal to 1, corresponding to CA class. In particular, the values of PC1, 2 and 4 increase its rating, while those of PC3 decreases it. On the contrary, in panel (B) an example of a patient with AS is shown, with a prediction equal to 0, corresponding to AS class: here only the value of PC4 and 5 increases its rating, while that of PC1, 2, 3, 6, 7 and 8 decreases it.

To conclude the explainability analysis, the class of the most important features in the different PCs was evaluated. Loadings of each PC, i.e., the coefficients describing the importance of the independent variables in each PC were considered and squared. Squared values, higher than half of the maximum squared loading, were included in the analysis and divided according to the feature class and finally summed up. Table 3 reports the sums of the considered squared PC loadings for each feature class. It can be noted that the first three PCs are mainly dependent on textural features while PC4 and 6 on FOS and the last three PCs on SS features.

**Table 3:** Relevance of the different feature class in the principal components (PCs): PCs loadings were squared and summed if the individual squared value is higher than half of the maximum squared loading.

PC	Feature class		
	SS	FOS	Textural
1	0.08	-	**0** **.** **51**
2	0.14	0.13	**0** **.** **31**
3	-	-	**0** **.** **55**
4	0.16	**0** **.** **19**	0.10
5	0.22	-	**0** **.** **35**
6	-	**0** **.** **25**	0.11
7	**0** **.** **32**	-	-
8	**0** **.** **26**	-	0.09
9	**0** **.** **30**	-	-

Bold values indicate the class with the highest squared loading for each PC.

## 4. Discussion

In the present study a robust and reliable radiomic-based pipeline was developed based on a dataset composed of patients with CA or AS, to differentiate LV hypertrophy due either of the two diseases. The pipeline included three steps of feature selection to obtain a set of (i) stable and discriminative, (ii) non-redundant and (iii) more relevant features, followed by the classification model development. A systematic analysis was performed in which, the influence of (i) multiple machine learning algorithms (kNN, SVM, DT, LR and GB), (ii) correlation thresholds (0.80, 0.85, 0.90, 0.95 and 1) and (iii) feature selection methods (*p*-value, LASSO, ssLASSO, PCA, ssPCA, SFS) were assessed. Specifically, with the data at disposal, the SVM algorithm, combined with a correlation threshold of 0.95 and PCA feature reduction method, outperformed all the other machine learning models.

Finally, an explainability analysis was conducted in order to gain insight in the trained model. In particular, the analysis revealed that the most impactful features were textural features, as observed from Table 3, which might reflect the differences existing between the hypertrophic phenotype caused by CA and AS. The increase in LV wall thickness caused by CA, is the result of an extracellular deposit of amyloid fibrils within the myocardium while the hypertrophy characterizing AS patients is given by the pressure overload on the LV. This difference seems to be captured by the radiomics analysis CA and AS differences in the hypertrophic structural pattern also appear to be partly detected by SS radiomic features.

From a clinical point of view, the main finding of the study is that radiomics can be used to differentiate CA from AS starting from CCT image scans whose acquisition is comprised in the interventional planning of AS patients undergoing TAVI. The gold standard for CA diagnosis is endomyocardial biopsy. However, endomyocardial biopsy is invasive, and some risks are associated with this technique. Nowadays, it is widely recognized that bone scintigraphy represents a reliable diagnostic tool for CA, in particular for the ATTR variant, avoiding endomyocardial biopsy. However, this diagnostic tool does not appear to be an optimal approach from logistic and economic standpoints (35). Regarding other imaging techniques, echocardiographic appearances seen in the advanced stages of CA are fairly pathognomonic (36) but most echocardiographic parameters do not provide CA diagnosis in the early stages of disease and do not to allow to distinguish CA from other restrictive or hypertrophic cardiomyopathy (37). CMR imaging offers a high-definition structural imaging and tissue characterization that are often incremental to information obtained on echocardiography. Some CMR markers, although pathognomonic in patients with biopsy-proven CA, are not specific for CA and can be elevated in other forms of cardiovascular disease, including reactive or replacement fibrosis and inflammation (38). Very recently, it has been shown that CMR-derived right ventricular global strains and various regional longitudinal strains provide discriminative radiological features for CA and hypertrophic cardiomyopathy differentiation (39). However, CMR is expensive and contraindicated in a substantial proportion of patients (e.g., patients with atrial fibrillation, advanced renal dysfunction or non-compatible intracardiac devices) (38).

Goto et al. (40) developed two deep learning algorithms using electrocardiogram (ECG) and echocardiography data coming from respectively 3 and 5 academic medical centers. The ECG-based model reached a mean C-statistic of 0.86 in differentiating 587 CA patients from 8,612 controls while the echocardiography-based model achieved better performances with a mean C-statistics of 0.95 in distinguishing 609 CA patients from 303 controls. Also, CMR imaging was employed in different studies: Zhou et al. (41) classified 139 patients (79 CA positive vs. 60 controls) by employing CMR radiomics-based machine learning algorithm with a mean accuracy of 80%, and Martini et al. (42) developed a deep learning model to diagnose CA in 206 patients achieving an accuracy of 88%.

As compared with the previous experience, several points of strength could be considered in our study. First, our results based on CCT radiomics obtained an accuracy of 93%, significantly higher as compared previous studies. Second, to the best of our knowledge, this is the first study to employing CCT for amyloidosis identification. This has several clinical implications. Indeed, several studies showed that CA is frequent (11.8%) (43) in patients with severe AS referred for TAVI and the challenge, in this context, is to differentiate a wooden horse (lone AS) from a Trojan horse (AS with CA) (35). Nitsche et al. (43) developed in this setting the RAISE (remodelling, age, injury, system, and electrical) score, to predict the presence of CA in patients with severe AS, obtaining a sensitivity and specificity of 84% and 94%, respectively. Alternatively, to RAISE score, Oda et al. (44) proposed the measurement of extracellular volume by using CCT dataset. However, this approach requires triple scan acquisition (unenhanced scan arterial phase acquisition and late scan) and higher volume of contrast agent. On the contrary our approach is easily performed by using the single arterial phase CCT dataset acquired during the usual diagnostic work-up of these patients.

There are a few limitations to this study that should be considered. Firstly, the population size was small, which means that these findings should be viewed as preliminary and confirmed with a larger dataset. A very different approach could be based on deep learning, however to the best of our knowledge, no pre-trained 3D deep learning model is available to consider the LV volume. Moreover the limited size of the study population does not allow appropriate transfer learning, necessary to adapt the pre-trained network. Secondly, the patients included in the study were randomly selected from a cohort of patients with AS and CA who were referred for CCT. Lastly, the study examined CA and AS separately and did not include patients who were affected by both conditions. Thus, these preliminary results have shown the radiomic features potential to distinguish CA from AS patients, but future studies will be needed both to confirm the results on larger dataset and to investigate the differences between lone AS and its coexistence with CA.

## 5. Conclusion

In this study, a radiomic-based machine learning model able to differentiate CA and AS patients was developed. The analysis investigated the effect that key choices, as the features selection pipeline and the machine learning algorithm, may have on the classification performance. These preliminary results show that radiomics might help in differentiating CA from AS using clinical routine available images.

Developing a computer-based application able to differentiate hypertrophic cardiac phenotypes given by diseases such as AS, versus those from CA, is clinically relevant as CA plays an important prognostic role and may adversely affect the prognosis of patients who are undergoing AVR surgery (either with traditional or transcatheter surgery, TAVI).

In addition, the current availability of drugs that improve the prognosis of patients with CA makes the correct and early detection of this clinical condition, which is often underdiagnosed and confused with other forms of cardiac hypertrophy, even more important.

Finally, once automatized the process of LV segmentation, this radiomic application has the potential to routinely detect sub-clinical AM from CT scans regularly acquired in clinical practise for TAVI planning.

## Data Availability

The raw data supporting the conclusions of this article will be made available by the authors, upon reasonable request.
