# Supplementary material for: A robust radiomic-based machine learning approach to detect cardiac amyloidosis using cardiac computed tomography
Source: Front Radiol. 2023 Jun 16;3:1193046. doi: 10.3389/fradi.2023.1193046 (PMC10426499; doi:10.3389/fradi.2023.1193046)
Supplement: Supplementary file 1 [file Datasheet1.docx]

Supplementary Material

A robust radiomic-based machine learning approach to detect cardiac amyloidosis using cardiac computed tomography

Francesca Lo Iacono^1^, Riccardo Maragna^2^, Gianluca Pontone^2,3^, Valentina D.A. Corino*^1,2^

*** Correspondence:** Valentina D.A. Corino: [valentina.corino@polimi.it](mailto:valentina.corino@polimi.it)

**Supplementary Table 1:** Image acquisition parameters for the cardiac computed tomography (CCT) images used in the studies. Numeric values are expressed as median and inter-quartile ranges.

| **CT IMAGE ACQUISITION PARAMETERS** | |
| --- | --- |
| **Tube Voltage (kVP)** | 120 [100-120] |
| **Tube current (mA)** | 506 [115-599] |
| **Slice thickness (mm)** | 0.625 [0.5-0.625] |
| **Pixel spacing (mm)** | 0.46 [0.43-0.52] |

**Supplementary Table 2:** Parameters values set for each classification model.

| **Classification model** | **Parameter** | **Value** |
| --- | --- | --- |
| **KNN** | Metric | Euclidean |
|  | Number of neighbors | 3 |
| **SVM** | Kernel | Polynomial function |
|  | Polynomial degree | 3 |
|  | Tolerance stopping criterion | 0.001 |
| **DT** | Criterion to split on node | Gini |
|  | Minimum samples split | 2 |
|  | Minimum samples leaf | 1 |
| **LR** | Loss function | Cross-entropy |
|  | Max iteration | 1000 |
|  | Tolerance stopping criterion | 0.0001 |
| **GB** | Loss function | Cross-entropy |
|  | Minimum samples split | 2 |
|  | Minimum samples leaf | 1 |
|  | Tolerance stopping criterion | 0.0001 |

KNN: k-nearest neighbors; SVM: support vector machine; DT: decision tree; LR: logistic regression; GB: gradient boosting

**Supplementary Table 3:** Sensitivity for the support vector machine (SVM) model as a function of the correlation threshold and the feature selection method. Each row represents a feature selection method, while each column represents a correlation threshold.

| **Feature selection method** | **Correlation threshold** | | | | |
| --- | --- | --- | --- | --- | --- |
|  | **0.80** | **0.85** | **0.90** | **0.95** | **1** |
| **p-value** | 0.93 | 0.87 | 0.87 | 0.93 | 1 |
| **LASSO** | 0.93 | 0.93 | 0.87 | 0.87 | 0.87 |
| **ssLASSO** | 0.93 | 0.93 | 0.93 | 0.93 | 0.93 |
| **PCA** | 0.93 | 0.93 | 0.87 | 0.93 | 0.93 |
| **ssPCA** | 0.93 | 0.93 | 0.93 | 0.93 | 1 |
| **SFS** | 0.93 | 0.80 | 0.87 | 0.87 | 0.93 |

LASSO: least absolute shrinkage and selection operator; ssLASSO: semi-supervised LASSO; PCA: principal component analysis; ssPCA: semi-supervised PCA; SFS: sequential feature selection.

**Supplementary Table 4:** Specificity for the support vector machine (SVM) model as a function of the correlation threshold and the feature selection method. Each row represents a feature selection method, while each column represents a correlation threshold.

| **Feature selection method** | **Correlation threshold** | | | | |
| --- | --- | --- | --- | --- | --- |
|  | **0.80** | **0.85** | **0.90** | **0.95** | **1** |
| **p-value** | 0.80 | 0.80 | 0.87 | 0.87 | 0.73 |
| **LASSO** | 0.80 | 0.73 | 0.80 | 0.80 | 0.67 |
| **ssLASSO** | 0.80 | 0.80 | 0.73 | 0.73 | 0.80 |
| **PCA** | 0.73 | 0.80 | 0.93 | 0.93 | 0.87 |
| **ssPCA** | 0.80 | 0.80 | 0.87 | 0.87 | 0.80 |
| **FFS** | 0.80 | 0.73 | 0.80 | 0.80 | 0.87 |

LASSO: least absolute shrinkage and selection operator; ssLASSO: semi-supervised LASSO; PCA: principal component analysis; ssPCA: semi-supervised PCA; SFS: sequential feature selection.

**Supplementary Figure 1:** Boxplots representing the average accuracy of the five machine learning models for the various feature selection methods: (A) least absolute shrinkage and selection operator (LASSO), (B) semi-supervised LASSO (ssLASSO), (C) principal component analysis (PCA), (D) semi-supervised PCA (ssPCA) and (E) sequential forwards selection (SFS). The average accuracy was obtained averaging all the correlation thresholds. kNN: k-nearest neighbors; SVM: support vector machine; DT: decision tree; LR: logistic regression; GB: gradient boosting.

*
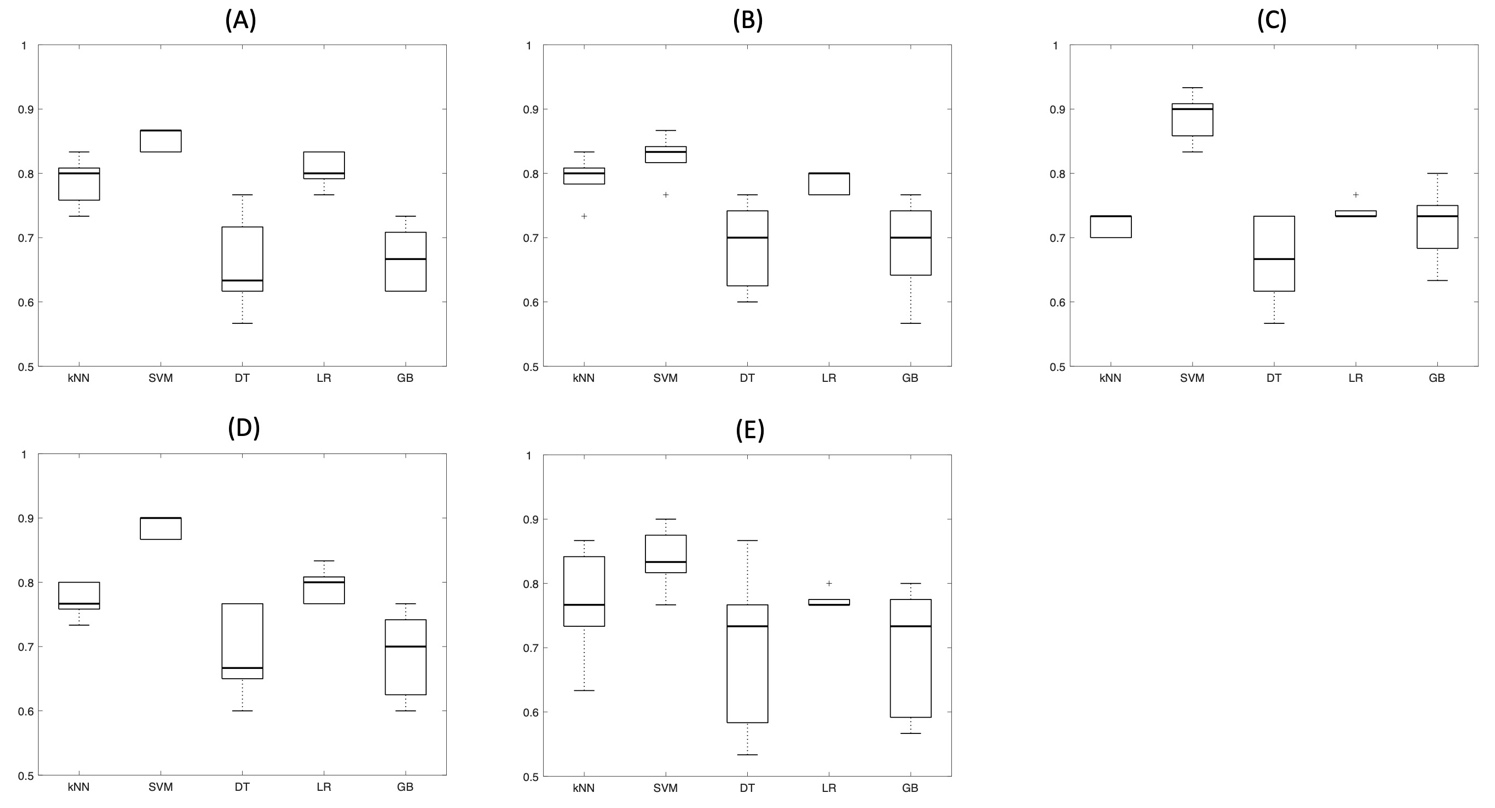
*

**Supplementary Figure 2:** Boxplots representing the average specificity of the five machine learning models for the various feature selection methods: (A) least absolute shrinkage and selection operator (LASSO), (B) semi-supervised LASSO (ssLASSO), (C) principal component analysis (PCA), (D) semi-supervised PCA (ssPCA) and (E) sequential forwards selection (SFS). The average accuracy was obtained averaging all the correlation thresholds. kNN: k-nearest neighbors; SVM: support vector machine; DT: decision tree; LR: logistic regression; GB: gradient boosting.

**Supplementary Figure 3:** Boxplots representing the average sensitivity of the five machine learning models for the various feature selection methods: (A) least absolute shrinkage and selection operator (LASSO), (B) semi-supervised LASSO (ssLASSO), (C) principal component analysis (PCA), (D) semi-supervised PCA (ssPCA) and (E) sequential forwards selection (SFS). The average accuracy was obtained averaging all the correlation thresholds. kNN: k-nearest neighbors; SVM: support vector machine; DT: decision tree; LR: logistic regression; GB: gradient boosting.
